# Functional Analysis of a Breast Cancer-Associated FGFR2 Single Nucleotide Polymorphism Using Zinc Finger Mediated Genome Editing

**DOI:** 10.1371/journal.pone.0078839

**Published:** 2013-11-12

**Authors:** Luisa J. Robbez-Masson, Csaba Bödör, J. Louise Jones, Helen C. Hurst, Jude Fitzgibbon, Ian R. Hart, Richard P. Grose

**Affiliations:** 1 Centre for Tumour Biology, Barts Cancer Institute – a Cancer Research UK Centre of Excellence, Queen Mary University of London, London, United Kingdom; 2 Centre for Haemato-Oncology, Barts Cancer Institute – a Cancer Research UK Centre of Excellence, Queen Mary University of London, London, United Kingdom; Imperial College London, United Kingdom

## Abstract

Genome wide association studies have identified single nucleotide polymorphisms (SNP) within *fibroblast growth factor receptor 2* (*FGFR2*) as one of the highest ranking risk alleles in terms of development of breast cancer. The potential effect of these SNPs, in intron two, was postulated to be due to the differential binding of cis-regulatory elements, such as transcription factors, since all the SNPs in linkage disequilibrium were located in a regulatory DNA region. A Runx2 binding site was reported to be functional only in the minor, disease associated allele of rs2981578, resulting in increased expression of FGFR2 in cancers from patients homozygous for that allele. Moreover, the increased risk conferred by the minor *FGFR2* allele associates most strongly in oestrogen receptor alpha positive (ERα) breast tumours, suggesting a potential interaction between ERα and FGFR signalling. Here, we have developed a human cell line model system to study the effect of the putative functional SNP, rs2981578, on cell behaviour. MCF7 cells, an ERα positive breast cancer cell line homozygous for the wild-type allele were edited using a Zinc Finger Nuclease approach. Unexpectedly, the acquisition of a single risk allele in MCF7 clones failed to affect proliferation or cell cycle progression. Binding of Runx2 to the risk allele was not observed. However FOXA1 binding, an important ERα partner, appeared decreased at the rs2981578 locus in the risk allele cells. Differences in allele specific expression (ASE) of FGFR2 were not observed in a panel of 72 ERα positive breast cancer samples. Thus, the apparent increased risk of developing ERα positive breast cancer seems not to be caused by rs2981578 alone. Rather, the observed increased risk of developing breast cancer might be the result of a coordinated effect of multiple SNPs forming a risk haplotype in the second intron of *FGFR2*.

## Introduction

Breast cancer is the most common malignancy among women, with an estimated 1 million new cases and over 400,000 deaths annually worldwide [1]. The development of breast cancers, in the absence of high penetrance susceptibility genes like *BRCA1* and *BRCA2*, is caused by a multitude of genetic factors, each conferring a small increase in the overall risk, and various environmental factors [2]. Genome wide association studies (GWAS) have successfully identified many risk loci linked with susceptibility to altered response to drug treatment and other phenotypic variations. Particularly, an haplotype of SNPs located in the second intron of the *Fibroblast growth factor receptor 2 (FGFR2)* gene has been linked to increased risk of ER positive breast cancer. This was one of the top five significant loci identified by early GWAS [3], [4], with an homozygous risk allele frequency of approximately 28% in the European population (ENSEMBL). However the connection between most of those variants, including the *FGFR2* haplotype, and the underlying mechanism of carcinogenesis remains unknown. Comprehensive functional validation studies are needed to better understand the biological significance of these risk alleles.

An early functional study on the *FGFR2* SNPs hypothesised that rs2981578 was the functional element of the risk haplotype, and that allele specific expression of *FGFR2* was mediated by differential binding of the trans-acting enhancer by the Runx2/Oct1 complex [5]. Since FGFR signalling, and FGFR2 in particular, has been implicated as a driving force in breast cancer [6], over-expression of *FGFR2* as a result of such alterations in transcriptional regulation was postulated as the underlying cause of the increased risk of developing breast cancer.

Given the vast genetic differences that exist between breast cancer cell lines [7], comparing different cell lines in terms of their SNP genotype is unworkable, and so we developed instead a set of isogenic breast epithelial cell line models to study the role played by rs2981578 in mediating breast cancer risk. To this end, zinc finger nuclease (ZFN) technology was used as a means of editing rs2981578 in breast cancer cells. This system relies on homologous recombination to create knock out and knock in models of genes in both organisms and cell lines, to study the role of genes and/or regulatory sequences. Random transgene integrations have the principal drawback of unpredictable gene expression due to multiple transgene copy integration and lack of control over integration sites [8], [9]. Site-specific recombination is much more precise, but has relatively low efficiency. A key advantage of targeted genome editing using ZFNs is that it leaves the neighbouring DNA intact and is therefore a more suitable approach for the study of regulatory DNA. Recently, ZFNs have been used to drive efficient genome editing in rat zygotes [10], human embryonic stem cells [11], human cancer cells [12] and human T cells [13]. A recent study has attempted to modulate the response to certain anti-cancer drugs by deleting polymorphisms in the pro-apoptotic gene *BIM*, which affect the response to tyrosine kinase inhibition [14].

In this study, ZFN technology was used as proof of concept to engineer and study functional intronic SNPs. Site specific genome editing was achieved using ZFN and homologous recombination, resulting in a panel breast cancer cell lines composed of three MCF7-derived clones heterozygous for rs2981578, and three MCF7-derived wild-type controls that lack the disease associated allele of the SNP.

## Experimental Procedures

### Cell Culture and Genomic DNA Isolation

The breast adenocarcinoma MCF7 cell line [15] and derived clones were cultured in DMEM supplemented with L-Glutamine and 10% foetal bovine serum (FBS), as were T47D, H3396, BT20, MDA-MB-231, MDA-MB-453 and β4-1089 [16] cell lines. MCF10A cells were cultured in DMEM:Ham’s F12 1:1 volume, insulin from bovine pancreas (10 µg/ml), Hydrocortisone (500 ng/ml), cholera enterotoxin (100 ng/ml), human EGF (20 ng/ml) and 5% horse serum (all from Sigma). ZR-75-1 and SKBR3 lines were cultured in RPMI medium (PAA laboratories) supplemented with 10% FBS. MDA-MB-468 cells required L15 medium and 10% FBS. SUM159 cells were cultured in Ham’s F12 medium with 5% FBS, insulin (0.01 mg/ml) and hydrocortisone (500 ng/ml).

Genomic DNA was purified from each cell line using the GenEluteTM mammalian genomic DNA miniprep kit (Sigma) according to the manufacturer’s instructions and the samples were sequenced using a Big Dye Terminator kit (Applied Biosystems).

### ERα Pathway Inhibition

Cells were seeded in 6 well plates at a density of 3×10^5^ cells per well in normal medium. The cells were treated with 1 µM Tamoxifen (Sigma) or with ethanol (vehicle control) for 48 hours and total RNA was purified using an RNeasy Kit (Qiagen) according to manufacturer’s recommendations. Complementary DNA was generated from 500 ng of RNA and quantitative real time PCR performed using SYBR Green (Invitrogen) and the StepOnePlus Real-time PCR system (Applied Biosystems). The following primers were used: hGAPDH_forward 5′-CAATGACCCCTTCATTGACC-3′; hGAPDH_reverse 5′- TTGATTTTGGAGGGATCTCG-3′; ERalpha_forward 5′GCACCCTGAAGTCTCTGGAA-3′; ERalpha_ reverse 5′TGGCTAAAGTGGTGCATGAT-3′; cMyb_forward 5′-GAAGGTCGAACAGGAAGGTTATCT-3′; cMyb_reverse 5′-GTAACGCTACAGGGTATGGAACA-3′; PS2_forward 5′-GAGAACAAGGTGATCTGCGC-3′; PS2_reverse 5′-TGGTATTAGGATAGAAGCACC-3′.

### FGFR2 Pathway Stimulation

Cells were seeded in 6 well plates at a density of 3×10^5^ cells per well in normal medium. After 24 hours, medium was replaced with starvation medium (DMEM +0.1% BSA). The following morning, starved cells were stimulated from 5 min to 1 hour with differing concentrations of ligand (100, 50, 10, 1 ng/ml of FGF7 or FGF10, Peprotech) and 300 ng/ml of Heparin (Sigma). At the end of the treatment time point, the cells were lysed in 2X NuPage Sample buffer (Invitrogen) supplemented with 10 mM DTT and western blotting was performed using anti phospho-ERK (#9101S, Cell Signalling) and anti-HSC70 antibodies (sc-7298, Santa Cruz).

### ER Positive Breast Tumours from Patients

Frozen tissue from ER positive breast tumours was obtained from the Breast Cancer Campaign Tissue Bank (Barts Cancer Institute, BCI), under ethical approval (Ethics REC reference: 10/H0308/49) from the North East London ethics committee. Total DNA from breast tissues was extracted using a GenEluteTM mammalian genomic DNA miniprep kit (Sigma) (according to manufacturer’s instructions) and total RNA was purified using Trifast reagent (PeqLab). SNP genotyping of rs2981578, rs1047100 and rs755793 was performed by Taqman SNP genotyping assay (Applied Biosystems). Genotyping results were visualized using the Genotyper software, version 1.0.1 (Applied Biosystems), whereas specific allele amplification data could be read using SDS software, version 2.3 (Applied Biosystems).

### *FGFR2* ZFN Pair

CompoZr™ custom made FGFR2 ZFNs were purchased from Sigma. Messenger RNAs encoding the two ZFN modules were generated from ZFN plasmids (linearised with XbaI) by run-off transcription using a MessageMax T7 mRNA transcription kit (Epicentre). The ZFNs were tested by transient transfection into MCF7 cells, to test for disruption of the sequence of intron 2 of *FGFR2*. Cutting efficiency at the target locus was determined by Surveyor (Cel I) endonuclease-based measurement of non-homologous end joining (NHEJ), as described [17], [18] (primers used in Cel I analysis: ZFN_For, 5′- GCAGAGTTTCTTGCCAGGTC-3′ and ZFN_rev, 5′- ACATTCCACGTTAAGAGCCG-3′). Analysis of off-target cleavage by ZFNs, which results in NHEJ-mediated indels, was performed by sequencing the top off-target hits as determined by the algorithm from the ZFN site website (http://ccg.vital-it.ch/tagger/targetsearch.html). The results are described in detail in Figure S1.

### ZFN-mediated Genome Editing of MCF7 Cells

ZFN pairs were transfected into MCF7 cells using the Amaxa System (Lonza). Nucleofection was performed using the Cell line NucleofectorTM kit L (Lonza) using the programme P-020. A repair template donor plasmid was constructed by cloning 2,154 base pairs of the *FGFR2* intron, corresponding to Chr10∶123,339,177-123,341,331, surrounding the SNP (G allele) at Chr10∶123,340,311 (GRCh37/hg19), into pJet1.2 (VWR International). 2×10^6^ cells were harvested with 10X Trypsin/EDTA (GE Healthcare) and resuspended in 100 µl complemented transfection solution, 2 µg of donor plasmid, 2 µg of pmaxGFP (Lonza) and 2 µg of each ZFN mRNA. Immediately after electroporation, 500 µl warm complete medium was added to the cuvette and the cell suspension was transferred to a 100 mm culture dish, with 10 ml warm complete medium. The medium was changed 24 h post-Nucleofection. GFP enrichment, using an ARIA II cell sorter (Becton Dickinson), was performed 48 hours post transfection, which constitutes the peak expression window for the pmaxGFP construct (Lonza). The cells were then seeded at a concentration of 400 cells/plate, in 150 mm diameter culture plates, and cultured for 14 days. Once the colonies reached approximately 100 cells in size, the medium was removed and the cells washed with sterile PBS. Individual colonies were picked and transferred to a 96 well plate for clonal expansion.

### FOXA1 Chromatin Immunoprecipitation

FOXA1 ChIP was carried as previously described [19] using 5 µg of anti-FOXA1 antibody (Ab5089, Abcam). Cells were plated in a 150 mm culture dish. After 24 h, test cells were deprived of oestrogen for 3 days by replacing the medium with phenol-red free DMEM (Sigma) supplemented with 5% charcoal-stripped FBS (Gibco). The starvation medium was changed every day for three days. The starved cells were then stimulated with 100 nM of β-oestradiol (Sigma) for 1 hour. The control plates were maintained either in full medium or starved without oestrogen stimulation. Real time quantitative PCR was used to assess the fold enrichment of FOXA1 binding at the rs2981578 locus. Primers binding the *Greb1* promoter were used as a positive control and primers recognising an intronic site of *Cyclin D1* with no FOXA1 binding site were used as negative control (CCND1_F, 5′-TGCCACACACCAGTGACTTT-3′; CCND1_R, 5′-ACAGCCAGAAGCTCCAAAAA-3′). A master mix was prepared as described previously and 2 µl of sample or input (1∶14 dilution) were added, in triplicate. The Ct values obtained were used to evaluate the total amount of DNA in samples and inputs. The enrichment was normalised first to the input and then to the negative control.

### Proliferation Assays

Cell viability, over a 72 h period, was measured by CellTiter 96 Aqueous One Solution Cell Proliferation assay (Promega). Cells were seeded in 96 well plates at a concentration of 2,500 cells/well, in triplicate for each time point (24 h, 48 h and 72 h). At the end of each time point, the medium was removed and replaced with 100 µl of fresh medium and 20 µl of CellTiter Solution. The plate was incubated at 37°C for 2 h. Absorbance was measured at 490 nm on an LT-4000 Microplate reader (Labtech). Wells without cells were used as blanks for normalisation.

For Ki67 staining, cells were plated on glass cover slips in 24 well plates at a density of 20,000 cells/well. The next day, cells were fixed in 4% paraformaldehyde (PFA, Sigma) at room temperature for 10 min and washed three times in PBS for 5 min. Cells were permeabilised in 0.1% Saponin (Sigma) for 10 min, followed by three PBS washes. Non-specific antibody binding was blocked by incubation for 1 h in 5% BSA in PBS, prior to incubation with anti-Ki67 antibody (FITC Mouse, 1∶100 dilution, BD Transduction). The cells were washed several times in PBS with one last wash in distilled water, before mounting on a glass slide with mounting medium (ProlongTM Gold DAPI antifade reagent, Invitrogen). DAPI (4′,6-diamidino-2-phenylindole), contained in the mounting medium, allowed fluorescent labelling of cell nuclei. Images were taken on a confocal laser-scanning microscope LSM 510 (Zeiss). Quantification was performed by counting the percentage of Ki67 positive cells per field of view, under 40x objective (10 fields were analysed for each cell clone).

### Cell Cycle Analysis

Cells at approximately 70% confluence were harvested by trypsination, pelleted and resuspended in 1 ml of cold 70% ethanol with vortexing. The cells were fixed at 4°C for 30 min before being processed for staining with propidium iodide (PI, Sigma). After two washes in PBS, the cells were resuspended in 350 µl of staining solution containing 50 µg/ml PI and 100 µg/ml RNaseA (Sigma) diluted in PBS. The tubes were protected from light and incubated at RT for 30 min.

The amount of DNA staining was assessed by flow cytometry using a FACSCalibur machine (BD Biosciences). Raw data were analysed using FlowJo™ software, using the Watson (Pragmatic) algorithm. Two-way Anova statistical test was used to determine significance (GraphPad Prism, version 5.03).

### Selection Pressure Experiment

MCF7 cells (2×10^6^ cells) were transfected in triplicate with mRNAs encoding the ZFN pairs, along with the MCF7 repair template, as described earlier. At passage 1 post-nucleofection, and every third passage thereafter, gDNA was extracted and used for Taqman SNP genotyping assay to determine relative presence of the major and minor allele of rs2981578 SNP over a period of 20 passages.

### Allele Specific Expression

Specific SNP genotyping assays (rs2981578, rs1047100 and rs755793, Applied Biosystems), using Taqman probes, were used to discriminate between homozygous and heterozygous SNPs in human breast tumour samples (using gDNA) and measure the amount of relative allele expression (using cDNA). Allele specific expression was measured in heterozygous samples only in order to measure the absolute ΔCt between each allele.

## Results

### Cell Line Editing of the *FGFR2* Breast Cancer Risk Haplotype

**Figure 1:**
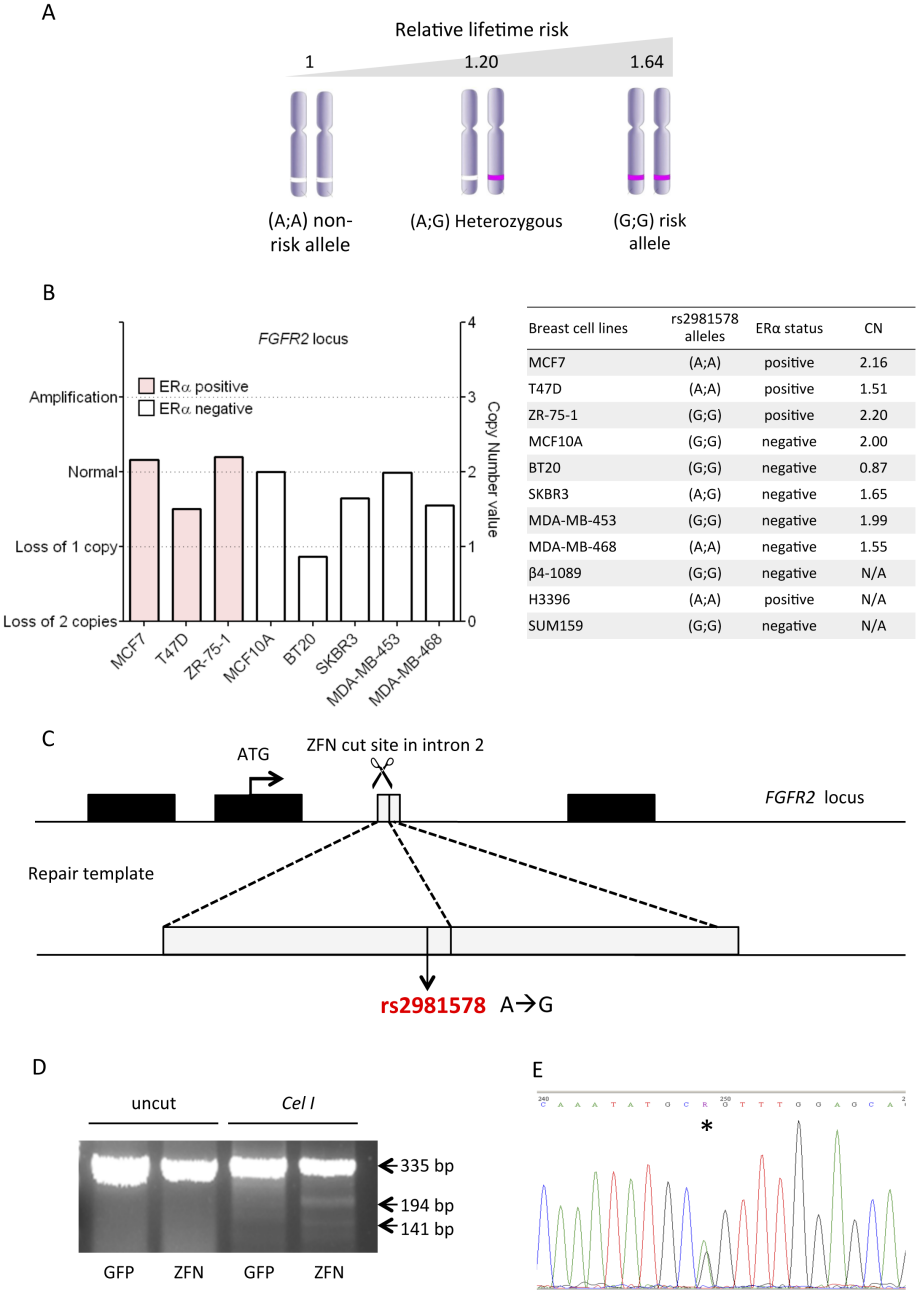
Analysis and editing of intron two of *FGFR2*. A) Estimated relative risk of rs2981578 associated with breast cancer development, for each possible genotype. Data from [3], [4]. B) Copy number variation (CNV) at the *FGFR2* locus in a panel of ERα positive (pink) and ERα negative (white) breast cancer cell lines. Data obtained from DNA copy number Affymetrix SNP 6.0 array, Cancer cell line Encyclopaedia (Broad Institute). The table represents the rs2981578 genotype in a panel of breast cancer cell lines and their respective ER status and *FGFR2* copy number (CN). C) The target site of the FGFR2 ZFN pair. Genome editing was carried out at in the second intron of *FGFR2*, at the ZFN cutting site, 100 bp away from rs2981578. An exogenous repair template was used for targeted homology repair and introduction of the risk allele in MCF7 cells. D) Surveyor Assay in MCF7 cells after ZFN or GFP transient transfection. Post PCR DNA products were digested with Cel-I endonuclease to assess ZFN-mediated cleavage of the target site. E) Sequencing trace of the rs2981578 locus (asterisk) showing the introduction of the risk allele (A;G) in the normally homozygous MCF7 cells (A;A).

Rs2981578 has three possible genotypes in diploid cells: (A;A), (A;G) and (G;G), where the G allele is the disease associated allele that confers an increased risk of developing ER positive breast cancer (Fig. 1A). One copy of the risk allele confers a 1.2 fold increase in risk for breast cancer development, and this figure increases to 1.64 for individuals homozygous for the risk allele (Fig. 1A) [4]. The SNP status of rs2981578 was investigated in several candidate breast cancer cell lines. Cell lines were classified dependent on their ERα status, since this was the only tumour characteristic found to be associated with *FGFR2* dependent risk, and their respective *FGFR2* copy number. Since many cultured cancer cell lines are highly aneuploid, it was important to identify lines that were diploid for chromosome 10, where *FGFR2* is located, in order to avoid having to target multiple *FGFR2* alleles. Copy number variation data from the Cancer Cell Line Encyclopaedia (Affymetrix SNP6.0 Array, CCLE, Broad Institute) were used to determine whether the candidate cell lines showed *FGFR2* deletion or amplification (Fig. 1B). All the cell lines investigated were homozygous, A;A or G;G, except SKBR3. The proportion of cell lines with the non-disease associated allele (four out of eleven A;A) was slightly lower relatively to the disease-associated allele (six out of eleven G;G). We hypothesised that the putative phenotype of rs2981578 could be more visible in the early stage of breast cancer development, rather than at a more advanced stage, where other oncogenic mutations might mask any phenotypes related to the SNP; therefore candidate cell lines that represented relatively early stage breast cancer, with only two copies of chromosome 10, were favoured. The MCF7 cell line, which is ERα positive and homozygous for the major, non risk-associated, allele of rs2981578, was chosen for ZFN-mediated genome editing.

Genome editing with engineered ZFNs relies on induction of targeted double-stranded break (DSB) by the nuclease heterodimer and by targeting the DSB in close proximity to the genomic locus of interest allows point mutations to be transferred with maximal efficiency from a repair template. Our ZFN pair introduced a DSB 100 bp 3′ to rs2981578 (Fig. 1C), located in the large second intron of *FGFR2*. To confirm the cutting efficiency of the ZFN pair, DNA from MCF7 cells transfected with mRNAs encoding the ZFN pairs was isolated and screened by surveyor assay. The success of ZFN targeting can be assessed directly by visualising the Cel-1 mediated digestion of heteroduplex products, formed by annealing wild type and ZFN targeted PCR products. This demonstrated significant cutting efficiency at the *FGFR2* locus (Fig. 1D).

Since modification of the rs2981578 risk locus entailed an extremely precise change, we designed a genome editing method without a drug selection strategy, to avoid altering the DNA sequence by anything more than the targeted SNP nucleotide (Fig. 1C). An exogenous donor construct comprising 1 kb homology on each side of the ZFN-targeted site and carrying the risk allele (G) of rs2981578, together with the ZFN mRNAs and pmaxGFP construct, were electroporated into MCF7 cells. SNP genotyping Taqman assay for rs2981578 was used to analyse 72 resultant individual single-cell clones, of which 3 clones (Het 1–3) were edited successfully and contained one *de novo* copy of the risk allele of rs2981578 (4.1% monoallelic genome editing efficiency), which was confirmed by sequencing (Fig. 1E). None of the clones screened showed biallelic modification of rs2981578. Three controls chosen from unmodified MCF7 clones (which were subject to the same treatment as heterozygous clones), and three heterozygous clones were selected for functional characterization. All these clones are freely available from the Grose Lab. Unfortunately, the generation of homozygous (G/G) clones proved extremely challenging and was beyond the scope of this study.

### Oestrogen Receptor Signalling and FOXA1 Binding in Heterozygous Clones

**Figure 2:**
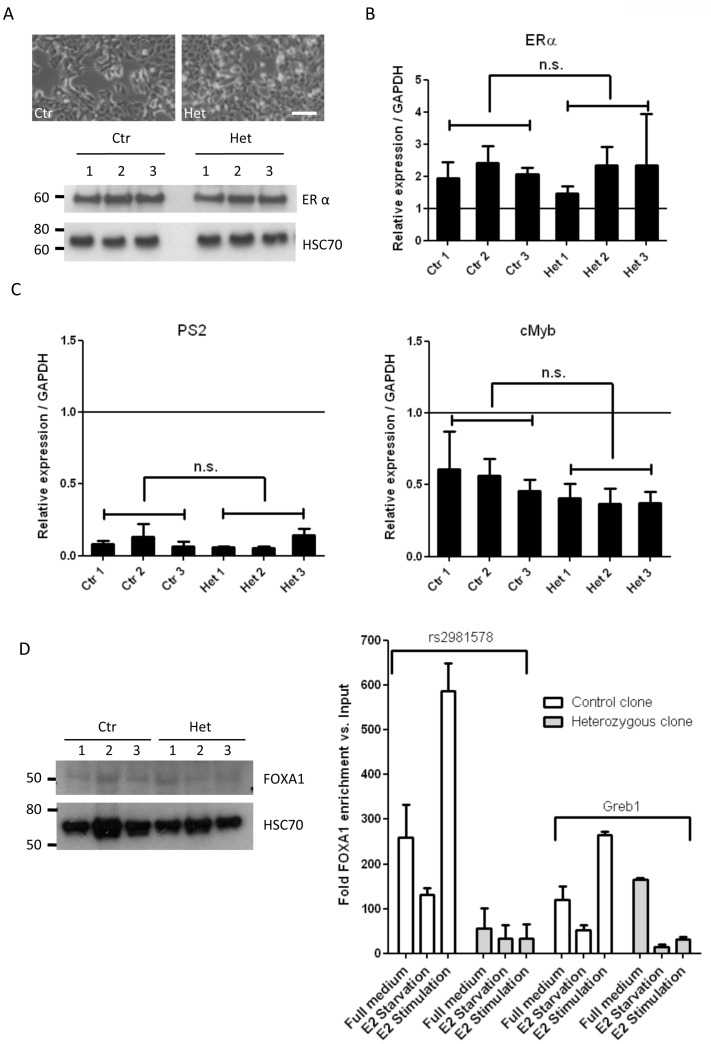
Functional impact of rs2981578 allelic modification in a panel of heterozygous and control MCF7 clones. A) Morphological appearance of a control versus a heterozygous clone (Scale bar: 50 µm) and expression of ERα in control and heterozygous clones. HSC70 was used as loading contol. Western blot is representative of three independent experiments. B) Quantitative RT-PCR of ERα expression level upon exposure to 1 µM Tamoxifen relative to control (vehicle, EtOH) for 48 h. C) Quantitative RT-PCR of PS2 and cMyb expression level, two target genes of ERα, upon exposure to 1 µM Tamoxifen relative to control (vehicle, EtOH) for 48 h. mRNA levels are shown relative to GAPDH expression, and normalized over untreated cells. Mean ± SEM of three independent experiments are presented. Two-way ANOVA showed no significant difference in expression levels between the control and heterozygous clones (ERα: p = 0.6491; PS2: p = 0.1098; cMyb: p = 0.2304). D) Expression of FOXA1 in the controls and heterozygous clones. HSC70 was used as loading control (western blot is representative of three independent experiments). FOXA1 ChIP in one control and one heterozygous clone following exposure to oestrogen (E2) (full medium, E2 starvation or E2 stimulation). Primers recognizing the rs2981578 locus and a positive control (*Greb1* promoter) were used. ChIP was performed in triplicate and the amount of precipitated DNA were normalized to the input DNA and a negative control (*CCDN1* intron). Student’s two-tailed t-Test was used to analyse significance.

MCF7 cells are a weakly metastatic breast cancer cell line, growing in clusters and retaining contact inhibition. At confluence, they show cobblestone morphology, typical of epithelial cell lines. There was no difference between heterozygous and control lines. The appearance of the sub-lines varied moderately between each other (Fig. 2A) but did not correlate with the rs2981578 genotype. Since ER positivity constitutes the only significant tumour characteristic that was associated with the *FGFR2* SNP haplotype, ERα expression levels in the MCF7 clones were determined. Western blot analysis showed no significant difference in the levels of ERα between the three heterozygous lines as compared to three control lines (Fig. 2A). To test whether the acquisition of one copy of the risk allele altered ER signalling in MCF7 clones, cells were treated for 48 hours with Tamoxifen, an ER antagonist, and the expression of ERα and two of its canonical target genes, *PS2* and *c-Myb* [20], [21] was examined. Tamoxifen treatment led to a significant increase in the levels of ERα mRNA in all clones, when compared to the untreated cells (Fig. 2B). We observed a significant reduction in the expression level of two of ERα target genes (p<0.0001), which was equivalent in both control and heterozygous clones. Since ER expression and responsiveness were similar in all the clones, we concluded that rs2981578 SNP status has no striking effect on ERα.

In a previous study, Runx2 was identified as the transcription factor mediating the increase in FGFR2 expression in cell lines with the disease associated allele of rs2981578 [5]. *In vitro* studies showed that exogenous Runx2 was able to bind the promoter of a *Iuciferase* reporter gene on a site containing multiple repeats of the disease associated allele and its surrounding sequence. The disease associated allele at the Oct1/Runx2 site stimulated transcription 2 to 5 fold over the non-disease associated allele, independently of orientation. ChIP data were less conclusive and showed only a relatively modest increase in Runx2 binding [5]. Our attempts to replicate the Runx2 ChIP data for the rs2981578 locus in MCF7 cells heterozygous for the risk allele failed to show any significant Runx2 binding at the SNP locus (data not shown). Therefore, we interrogated publicly available online whole-genome ChIP-seq data, to identify other potential transcription factors capable of binding at the rs2981578 locus. Data from MCF7 and HepG2 cell lines revealed that the pioneer factor FOXA1 binds to DNA at this locus. FoxA1 is responsible for opening condensed chromatin, facilitating access by other transcription factors, and has been shown to play an important role in maintaining euchromatic conditions and to be required for ERα binding [19]. Thus FOXA1 constituted an ideal candidate for studying the link between *FGFR2* intronic SNPs and increased risk of ER-positive breast cancer. The binding of FOXA1 to the rs2981578 SNP locus was confirmed in MCF7, T47D and ZR75-1 cell lines by ChIP-seq data analysis from a study on FOXA1 and ERα function in breast cancer [22]. Since FOXA1 is capable of binding transcriptionally inactive chromatin, MCF7 clones were either cultured in full medium or starved of oestrogen for 4 days and stimulated (or not) with 100 nM of β-oestradiol for 1 hour, prior to chromatin isolation and ChIP analysis. Sites within the fourth intron of *CCND1* (Cyclin D1) and the *Greb1* (growth regulation by oestrogen in breast cancer 1) promoter were used as negative and positive control, respectively, for FOXA1 binding [23]. As expected, control cells showed enhanced binding of FOXA1 to the *Greb1* promoter following oestrogen stimulation (additional 200 fold FOXA1 enrichment versus input compared to starvation conditions (p = 0.005)). Heterozygous cells showed relatively lower enrichment of FOXA1 binding. Despite an unexpected high level of FOXA1 binding to the *Greb1* locus in heterozygous cells growing in full serum, the cells still showed a positive response of FOXA1 binding to the *Greb1* promoter following oestrogen stimulation (additional 14 fold FOXA1 enrichment versus input compared to starvation conditions (p = 0.02), Fig. 2D). Control clones (A;A) showed significantly enhanced FOXA1 binding at rs2981578 relative to heterozygous clones (A;G) in all culture conditions, but most notably following ERα stimulation (p = 0.002). Total FOXA1 levels were equal in both control and heterozygous cell lines (Fig. 2D).

### FGFR Signalling in ZFN-modified Clones

**Figure 3:**
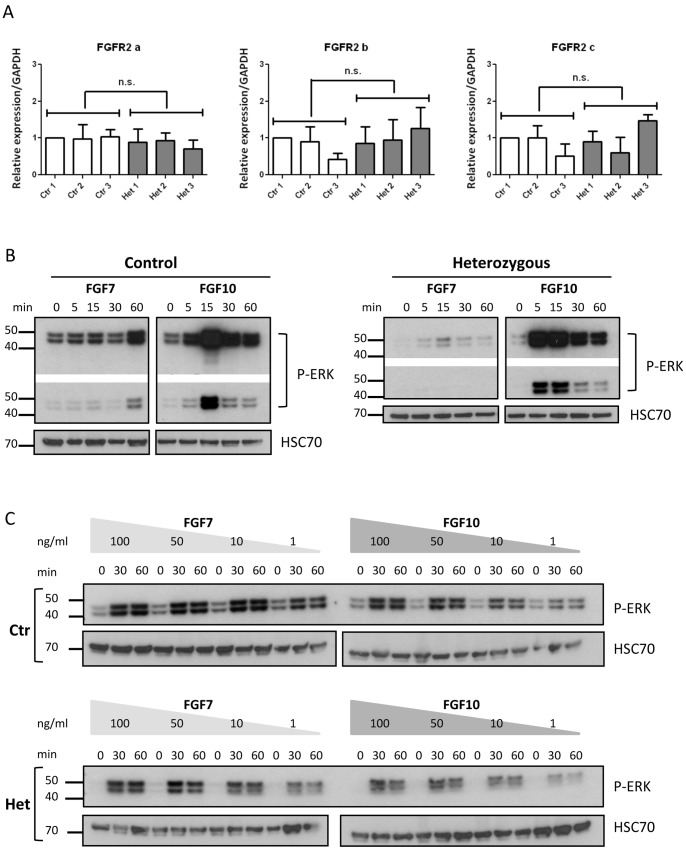
FGFR2 expression and signaling. A) Quantitative RT-PCR of FGFR2 isoforms (FGFR2a, FGFR2b and FGFR2c) in control and heterozygous clones. Error bars represent SEM of three independent experiments. Two-way ANOVA showed no statistical significance in expression of the receptors in control versus heterozygous clones. B) Representative western blots of ERK phosphorylation following stimulation of control and heterozygous clones with 100 ng/ml FGF7 and FGF10. HSC70 is used as loading control. C) Representative western blots of ERK phosphorylation following stimulation of the MCF7 clones with different amount of FGF7 and FGF10. HSC70 is used as loading control. The stimulations were performed in triplicate.

Having determined that rs2981578 status may determine levels of FOXA1 binding, we investigated FGFR2 receptor expression and signalling in the control and heterozygous MCF7 clones. They expressed all FGFR2 isoforms, predominantly the epithelial-associated isoform FGFR2-b (Ct value = 32 for FGFR2b vs. 34–35 for FGFR2a and c). Real-time PCR showed no statistically significant difference, in terms of isoform levels, between the control and the heterozygous clones (Fig. 3A). Note that the three isoforms are all expressed relative to GAPDH, but not to each other. The same clones shown in figure 2E were used in cell-based assays. Firstly, cells were stimulated with either 100 ng/ml FGF7 or FGF10, in the presence of heparin. Both FGF10 and FGF7 elicited robust ERK phosphorylation, sustained after 60 minutes of stimulation in both control and heterozygous cells (Fig. 3B). The sensitivity of the receptors to ligand concentration was also assessed (Fig. 3C). Even the smallest amount of ligand (1 ng/ml) elicited ERK phosphorylation, demonstrating no apparent change in receptor affinity for the ligands. From these first observations, the risk allele of rs2981578 did not affect directly the expression level or the signalling of FGFR2.

### Assessing Allele Specific Effects on Cell Proliferation *in vitro*

**Figure 4:**
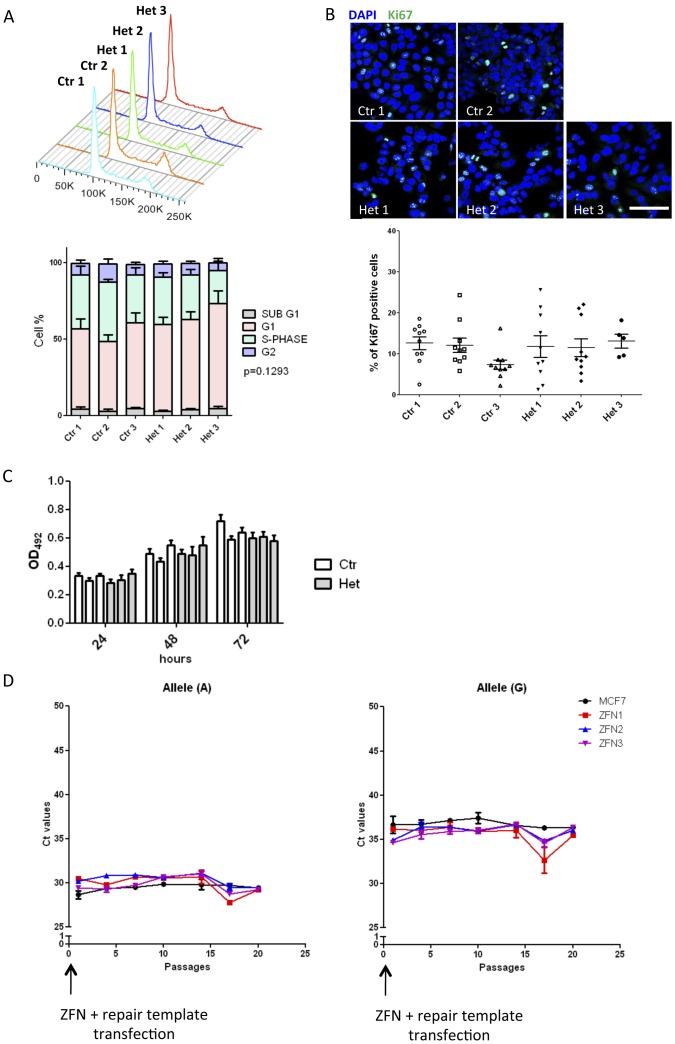
Investigating allele-specific effects on cell proliferation. A) Cell cycle analysis by PI staining and flow cytometry. No statistical differences were observed between the proportion of cells in each phase of the cell cycle between the control and the heterozygous clones (2-way ANOVA p = 0.1293). Error bars represent SEM of three independent experiments. B) Ki67 staining of the fixed MCF7 clones was performed to assess cell proliferation. Quantification was performed by counting the percentage of positive cells in 10 fields of view for each clone (on average 976 cells/10 fields). Mean ± SEM of three experiments are represented. One-way ANOVA showed no significant difference in proliferation between clones (p = 0.3573). Scale bar = 50 µm. C) MTS assay comparing the cell number between control and heterozygous clones over a period of 72 hrs. Mean ± SEM of three experiments are presented. D) Three independent cultures (ZFN1, ZFN2 and ZFN3) of wild-type MCF7 cells were transfected with ZFN mRNA and the exogenous repair template (containing the risk allele) and kept in culture over a period of 20 passages. The amount of each allele of rs2981578 was assessed every three passages using a specific SNP genotyping Taqman assay. The results are represented as Ct values for each allele over time. Untransfected MCF7 cells were used as controls.

In order to detect the impact of the single nucleotide change, heterozygous MCF7 clones were compared to their control counterparts in a series of *in vitro* assays. Firstly, the six clones were subjected to cell cycle analysis using PI staining followed by flow cytometry. The heterozygous clones displayed a normal cell cycle profile, similar to the wild-type controls (Fig. 4A). All clones showed reduced proliferation rate compared to MCF7 cells that had not been subject to single cell cloning (data not shown). Anti-Ki67 staining (Fig. 4B) and MTS assay (Fig. 4C) did not reveal any significant differences in proliferation between the clones. The process of single cell cloning did not affect the proliferative capacity of MCF7 cells (data not shown).

In order to test whether SNP genotype may influence the proliferation rate of cells that had not undergone single cell cloning, three separate flasks of MCF7 cells were transfected with ZFN mRNA risk allele repair template, and maintained as a polyclonal population. The three heterogeneous populations (ZFN1, ZFN2, ZFN3), composed of a mixture of wild-type MCF7 (A;A) and ZFN-modified cells (A;G or G;G), were cultured over a period of 20 passages. The relative frequency of each rs2981578 allele was measured over time using allele-specific Taqman probes to monitor any changes in the proportion of the two different genotypes.

The Ct values revealed, as expected after ZFN genome editing, a predominance of wild-type cells (with Ct values around 30 cycles), with a slight increase (2 cycles difference) in G allele frequency post ZFN transfection, that persisted for 3 passages (Fig. 4D). However, the Ct values returned to the level of the control, untransfected cells rapidly and no additional changes in Ct values were observed. The apparent increase in G allele frequency at passage 17 was an artifact caused by the poor quality of the genomic DNA samples, as this drop in Ct values was observed for both G and A alleles. Thus the presence of the G allele in the *FGFR2* haplotype did not give a measurable growth advantage to rs2981578 modified MCF7 cells in 2D culture.

### FGFR2 Allele Specific Expression in a Panel of Breast Cancer Samples

**Figure 5:**
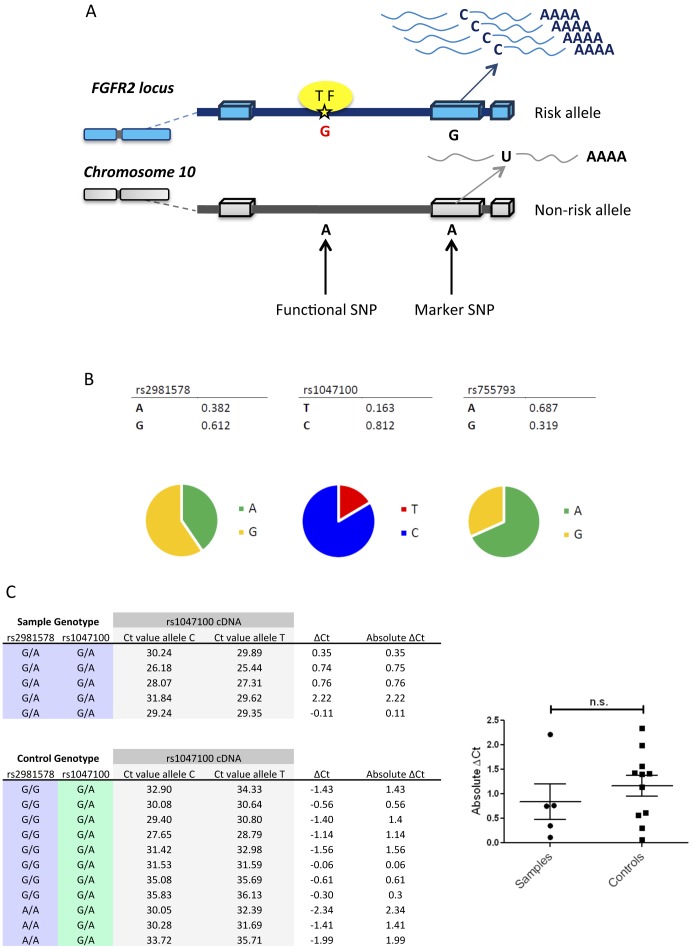
Assessment of ASE in breast cancer. A) Cartoon representing ASE where a cis-regulatory difference exists between G (blue) and A (grey) alleles. The activity of allele G is higher because of the differential binding of a transcription factor (yellow), which results in a relative abundance of blue mRNA transcript. A marker SNP, located in the coding region of the gene and with a heterozygous genotype, is used to differentiate the origin of each mRNA transcript synthesized (C and U). B) Allele frequencies for rs2981578 and two marker SNPs measured in a panel of 72 ERα positive breast cancer samples. C) Absolute ΔCt was measured in five samples that were heterozygous both for rs2981578 and rs1047100 (marker SNP) and compared to 11 controls in which rs2981578 was homozygous. Mann Whitney test revealed no statistical differences between the two groups.

Using the relative expression levels of variant SNP alleles within the coding region of a gene in the same sample (instead of using total mRNA levels originating from the two different copies of a gene) is an effective approach for identifying cis-acting regulatory SNPs [24]. Since rs2981578 is intronic, and therefore spliced out of mature mRNA, the allelic origin of each mRNA molecule was tracked by looking at additional heterozygous marker SNPs in the coding region (Fig. 5A).

Potential marker SNPs located in the coding region of *FGFR2* were identified using the Ensembl Genome Browser website [25], by looking at the single nucleotide variants observed in the different FGFR2 transcripts. Among 327 total variations found in the coding sequence, 148 were synonymous variants and 179 were non-synonymous. Two of those variants were shortlisted, since they showed minor allele frequencies greater than 10%. The essential characteristic of a marker SNP is its heterozygosity, thus minor allele frequency is an important factor because the greater the minor allele frequency, the better the chance of identifying heterozygous samples within cell lines or patient tissue samples.

SNP rs1047100 was identified as a synonymous SNP located in exon six of *FGFR2* (GTA/GTG). This nucleotide variance at position Chr10∶123298158 (GRCh37) encodes for valine in both cases. The minor allele (A) frequency varies between 8% to 22% in the different populations of the 1000 Genomes project [26]. The second marker was the non-synonymous SNP rs755793 (ATG/ACG) in exon five, Chr10∶123310871 (GRCh37). The ancestral codon, containing the thymine nucleotide, encodes for a methionine, substituted for threonine in the presence of the C allele. The minor allele (C) frequency varies greatly between populations, with a 36% frequency in African populations and an absence in European populations. Therefore, SNP rs1047100 was used predominantly in this study to determine the allelic origin of the FGFR2 mRNA molecules, because of the more homogeneous allele frequencies across populations and the fact that this change does not affect the amino acid sequence of the protein translated from the mRNA transcript.

Given the established limitation of using cell lines (too few in number and not carrying the adequate genotypes) (Fig. 1C), tissues from patients with ERα positive breast cancer were interrogated. Breast tissue samples were obtained from the Breast Tissue Bank at Barts in collaboration with Prof Louise Jones (ethics approved ref no. 05/Q0403/199) and selected purely on the basis of ERα positivity, regardless of treatment and ethnicity (figure S2). DNA and RNA from 72 ERα positive breast tumours and their surrounding tissues were used and each sample was genotyped for rs2981578 and the two marker SNPs (Fig. 5B). The allele frequencies of rs2981578 and rs1047100 in the patient samples were representative of the overall population data from the 1000 Genomes project (data not shown). Allele G of rs755793 was represented at a frequency higher than predicted from population data, indicating a potential bias towards an increased number of patients with African descent in the sample set. However, only 8.3% of the patients were of a Black background compared to 68% of a White background. Additionally, patients qualified as Asian in the sample set (composed of Indian, Bangladeshi and Pakistani patients) represented 10% of the samples and were not representative of the East Asian population (ASN) of the HapMap or the 1000 genomes data bases, composed mostly of Chinese, Japanese and Vietnamese individuals. Little information is available as yet on SNP allele frequencies in Indian, Bangladeshi and Pakistani populations (SAN, south Asian super population code).

Five samples, which were heterozygous for both functional and marker SNPs, were selected for ASE analysis (Fig. 5C). Real time PCR using allele-specific Taqman probes was performed for each sample, using complementary DNA (cDNA) templates. Imbalanced allelic expression is detected when the heterozygous allele ratio in mRNA (cDNA) differs from the normal allelic ratio of 1∶1. Cycle threshold (Ct) values obtained for both alleles of rs1047100 in cDNA were subtracted to obtain the absolute differences between Ct values (ΔCt) (Fig. 5C). Mann Whitney test indicated that the results did not show significant difference in absolute levels of expression (i.e. allelic imbalance) in the heterozygous samples compared to controls (p = 0.1645)(A;A and G;G genotypes).

## Discussion

GWAS have shown that the SNP haplotype in intron two of *FGFR2* is an important risk locus for the development of breast cancer [3], [4], but they do not address the mechanisms underlying risk association. We have used a novel genome editing approach to address the translational relevance of these data.

Conventional methods for the study of gene function can be challenging when looking at non-coding DNA regulatory sequences. Commonly, indirect methods such as Luciferase assay are used, but do not include all the cellular factors that might influence gene expression regulation (eg. endogenous trans-acting factors, epigenetic marks, chromosome conformation). ZFN-mediated genome editing presents several advantages over conventional methods as it can generate isogenic cell lines in which modifications at the endogenous genomic DNA sequence have been introduced without any additional changes in the DNA sequence. The biological variability associated with the use of different human cancer cell lines might thereby be abrogated and the study of single polymorphisms in identical genetic context made possible. Here we show that ZFN genome editing can be used in the study of cancer polymorphism risk factors.

We obtained three MCF7 clones carrying one copy of the rs2981578 risk allele (none had a biallelic change) and three other non-modified clones were selected as controls. The potential off-target effects of the FGFR2 ZFN pair were evaluated by sequencing of the top seven putative off-target binding sites, and no deletions due to NHEJ were observed in any of the clones. There were no discernible SNP dependant differences in the appearance of any of the MCF7 clones, although the cell lines did vary. It has often been reported that MCF7 cells, like many cell lines [27] have a tendency to deviate from their initial phenotypes as the number of passages in culture increases, and discrepancies in phenotypic appearance may also have been exacerbated by the stress of single cell cloning.

*FGFR2* has been reported to act as an oncogene in breast cancer and increased FGF signalling might promote cancer initiation or progression by protecting the cells from apoptosis [28] and stimulating growth and proliferation [29]. Cell-based assays showed that there was no change in cell cycle progression, nor any apparent advantage in cell growth in cells carrying the risk allele of rs2981578 (heterozygous versus non-modified controls). Crucially, it was established that Runx2 was not the key transcription factor mediating the rs2981578 risk, but instead, the pioneer factor FOXA1 appears more important. FOXA1 ChIP showed a reduced binding of FOXA1 to the SNP locus in two out of three of the heterozygous clones, whereas a very strong binding was observed in two out of three control cell lines. FOXA1 is crucial in mediating the binding of ERα to its target genes, and whole genome ChIP-seq screening has demonstrated that FOXA1 plays a role in the reprogramming of ERα binding sites during breast cancer progression [23], [30]. Interestingly, Ross-Innes and colleagues (2012) have shown that ERα binding is a dynamic process and that new ERα-binding sites were unique to seven patients with poor outcome as compared to eight patients with good outcome. When using the ChIP-seq data from that study, ERα was bound a few hundred base pairs away from the rs2981578 locus and only in samples associated with poor outcome. The current hypothesis regarding the role of FOXA1 in breast cancer is that FOXA1 is capable of mediating a reprogramming of the ERα binding site [23]. The role of each individual SNP forming the *FGFR2* haplotype, or their collective effect, on the dynamics of FOXA1 binding at the *FGFR2* locus remains to be elucidated.

The cohort of 72 patient samples did not show any allelic imbalance in FGFR2 expression. However, the heterogeneous nature of the tumour samples used might explain the lack of allelic imbalance if ASE is cell type specific. Indeed, published data suggest that the rs2981578-associated risk is cell type-dependent, and that the increased FGFR2 signaling and resulting oncogenic phenotype was only observed in stromal fibroblasts and not in cells of epithelial origin, like the MCF7 cell line [31]. It was also reported that the phenomenon of ASE is not present in 100% of heterozygous individuals and that other heritable factors might determine whether or not an allele is differentially expressed, indicating that an increased cohort of patients would be required to gain more statistical power to determine ASE [32]. Interestingly, the ethnic composition of our patient cohort has revealed that genetic data on population originating from central and western Asia, such as India, Bangladesh and Pakistan, are currently missing from the main publicly available databases such as the 1000 Genomes project (Figure S2).

Several limitations were encountered during the genome editing process, limiting the number of clones available for screening. One of the major obstacles was choice of potential ZFN binding sites for the SNP editing. The ZFN target sequence had to be restricted to the immediate vicinity of the target SNP, which meant that the optimal ZFN pair was less efficient than if the whole *FGFR2* locus been available for targeting. The problem of relative low efficiency of gene editing is common to many other studies and a lot of efforts are now being put into improving ZFN technology, as exemplified by recent reports suggesting the use of the proteasome inhibitor MG132 during the editing process as a way to increase the half-life of ZFN proteins [33], or the use of surrogate reporters that express GFP only when the reporter has been cleaved by the ZFN and a consequent frame shift mutation has occurred [34].

Taken together, we have shown that, while the SNP status of a cell line can be engineered specifically at the nucleotide level, in the case of rs2981578, this has no clear effect on cell phenotype. To complement our *in vitro* studies, we have analysed a panel of clinical samples for ASE, but again there is no clear evidence for rs2981578 status impacting of FGFR2 expression. Since the data implicating the *FGFR2* intron 2 haplotype in breast cancer are clear, from many independent studies, we hypothesise that there must be alternative SNPs impacting on cell behaviour.

## Supporting Information

Figure S1 **Assessment of FGFR2 ZFN off-target effect.** A) Potential off target sites as determined from the ZFN site website (http://ccg.vital-it.ch/tagger/targetsearch.html). When a nucleotide mismatch is found at a given position between query and hit, the mismatched position is highlighted and underlined; the original nucleotide being displayed underneath (red). The spacer sequence size is represented by Ns (green). Results also show the number of mismatches between queries and mismatch site, and the genomic locus of the putative off-target site. B) Sequencing results of the off-target ZFN binding site for each clone. A tick means that the sequence was identical to the Ensembl database, proving that the ZFN did not cut that locus. N.A. refers to a sequencing reaction that failed to give readable sequencing trace.
(TIF)

Figure S2 **Ethnicity of breast cancer samples.** Proportion of each ethnicity within the 72 breast cancer samples obtained from the Barts Breast Tissue Bank.
(TIF)
