# Supplementary figures and images for: Functional Analysis of a Breast Cancer-Associated FGFR2 Single Nucleotide Polymorphism Using Zinc Finger Mediated Genome Editing
Source: PLoS One. 2013 Nov 12;8(11):e78839. doi: 10.1371/journal.pone.0078839 (PMC3827080; doi:10.1371/journal.pone.0078839)

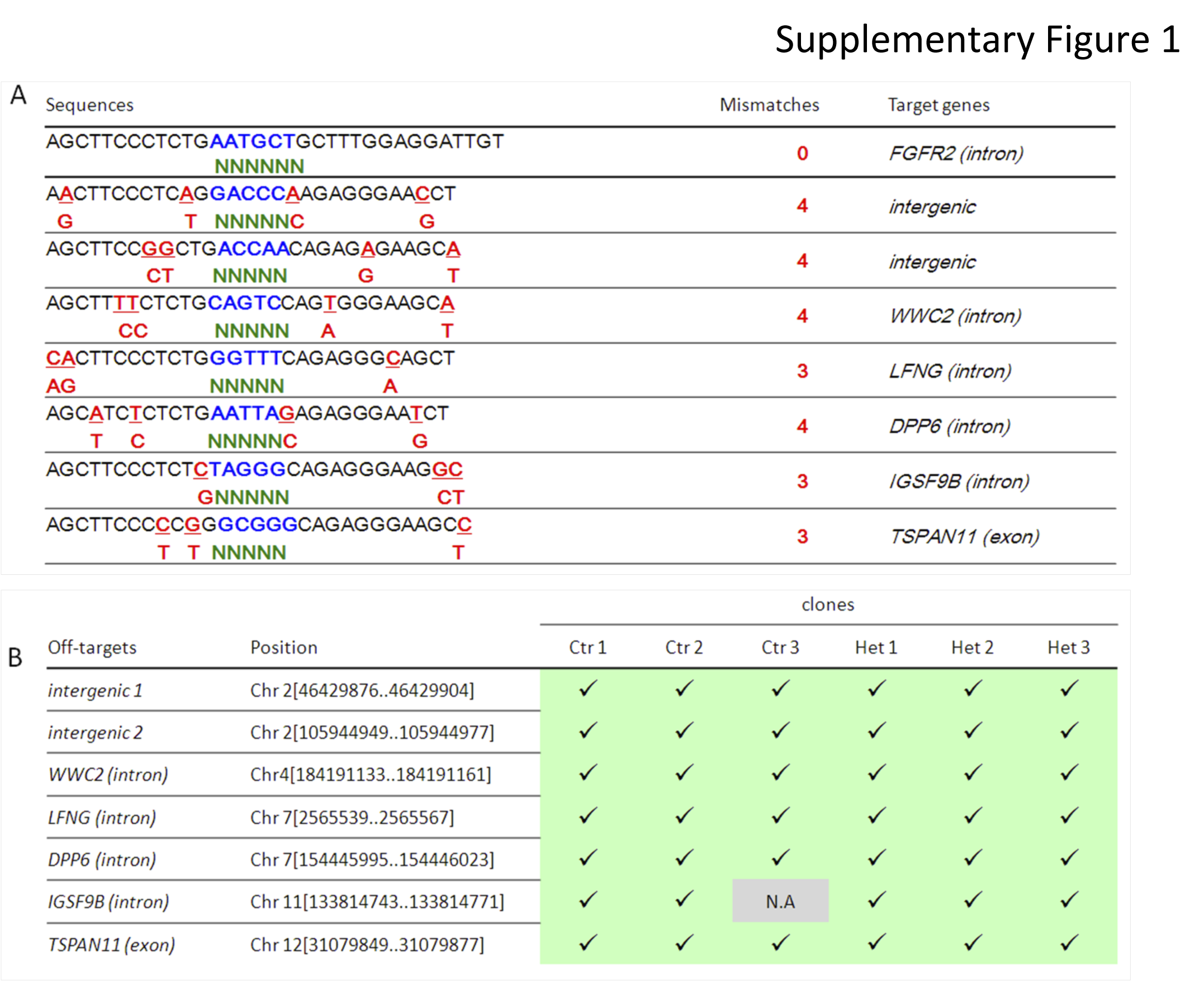

Supplement: Figure S1 — Assessment of FGFR2 ZFN off-target effect. A) Potential off target sites as determined from the ZFN site website (http://ccg.vital-it.ch/tagger/targetsearch.html). When a nucleotide mismatch is found at a given position between query and hit, the mismatched position is highlighted and underlined; the original nucleotide being displayed underneath (red). The spacer sequence size is represented by Ns (green). Results also show the number of mismatches between queries and mismatch site, and the genomic locus of the putative off-target site. B) Sequencing results of the off-target ZFN binding site for each clone. A tick means that the sequence was identical to the Ensembl database, proving that the ZFN did not cut that locus. N.A. refers to a sequencing reaction that failed to give readable sequencing trace. (TIF) [file pone.0078839.s001.tif]

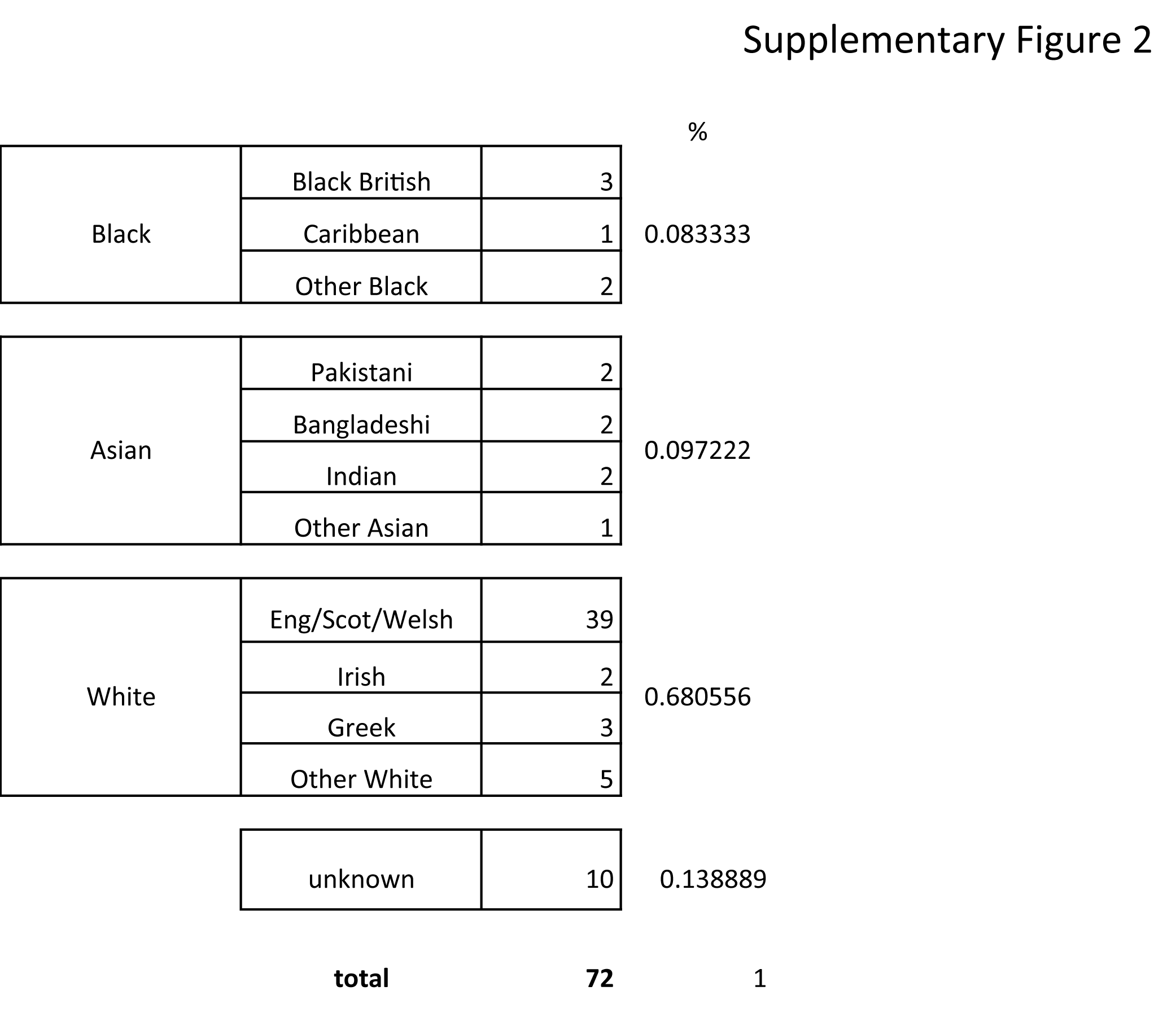

Supplement: Figure S2 — Ethnicity of breast cancer samples. Proportion of each ethnicity within the 72 breast cancer samples obtained from the Barts Breast Tissue Bank. (TIF) [file pone.0078839.s002.tif]
